# Effects of type I *Diacylglycerol O-acyltransferase* (*DGAT1*) genes on soybean (*Glycine max* L.) seed composition

**DOI:** 10.1038/s41598-021-82131-5

**Published:** 2021-01-28

**Authors:** Sepideh Torabi, Arjun Sukumaran, Sangeeta Dhaubhadel, Sarah E. Johnson, Peter LaFayette, Wayne A. Parrott, Istvan Rajcan, Milad Eskandari

**Affiliations:** 1 Department of Plant Agriculture, University of Guelph, Guelph, ON Canada; 2 London Research and Development Centre, Agriculture and Agri-Food Canada, London, ON Canada; 3 Department of Biology, University of Western Ontario, London, ON Canada; 4 Center for Applied Genetic Technologies, University of Georgia, Athens, GA USA; 5 Department of Crop and Soil Sciences, University of Georgia, Athens, GA USA

**Keywords:** Biotechnology, Genetics, Plant sciences

## Abstract

Type I Diacylglycerol acyltransferase (DGAT1) catalyzes the final step of the biosynthesis process of triacylglycerol (TAG), the major storage lipids in plant seeds, through the esterification of diacylglycerol (DAG). To characterize the function of *DGAT1* genes on the accumulation of oil and other seed composition traits in soybean, transgenic lines were generated via trans-acting siRNA technology, in which three *DGAT1* genes (Glyma.13G106100, Glyma.09G065300, and Glyma.17G053300) were downregulated. The simultaneous downregulation of the three isoforms in transgenic lines was found to be associated with the reduction of seed oil concentrations by up to 18 mg/g (8.3%), which was correlated with increases in seed protein concentration up to 42 mg/g (11%). Additionally, the downregulations also influenced the fatty acid compositions in the seeds of transgenic lines through increasing the level of oleic acid, up to 121 mg/g (47.3%). The results of this study illustrate the importance of *DGAT1* genes in determining the seed compositions in soybean through the development of new potential technology for manipulating seed quality in soybean to meet the demands for its various food and industrial applications.

## Introduction

Soybean (*Glycine max*) is an economically valuable crop across the world because of its significant contribution to the worldwide vegetable oil consumption and protein meal supplies^1^. While increasing the level of both oil and protein contents in high-yielding soybean varieties is desirable, the simultaneous improvement of the two traits has been challenging due mainly to their quantitative genetic architectures, their negative inter-relationship, as well as their effects on other important agronomic traits, including yield^1–3^. While the concurrent improvement of soybean seed oil and protein contents has been challenging using conventional phenotypic selection methods and classical molecular breeding tools^4–6^, advanced genetic engineering procedures have provided potential tools for overcoming the dilemma of modulating one pathway without disrupting others^7^.

Plant seed oils, including soybean seed oil, are mainly composed of triacylglycerol (TAG)^8,9^. TAG, which is neutral lipids and stored in the cytosolic lipid droplet of plant seeds, are main constituents of seed oils and the major storage lipids. TAG is essential sources of energy and metabolic substrates for seed germination, seedling growth, pollen development, and sexual reproductions in many plant species^10–12^.

**Figure 1:**
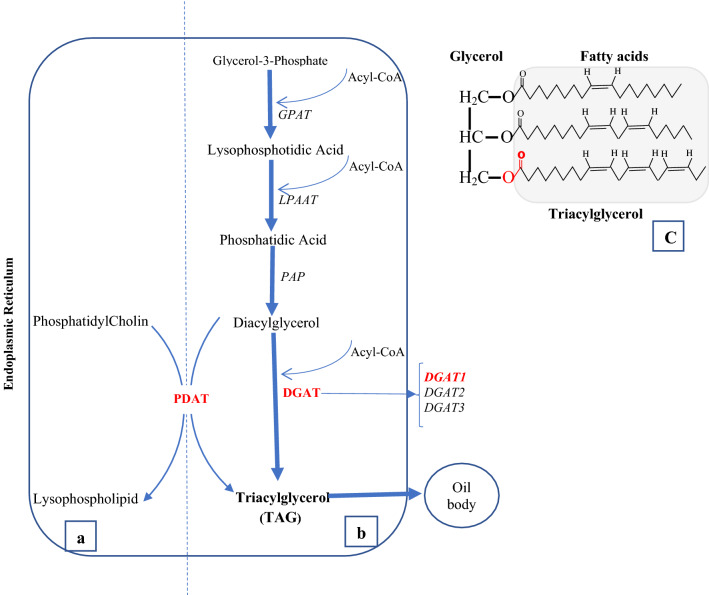
A schematic representation of TAG production. (**a**) acyl-CoA independent acylation. (**b**) acyl-CoA dependent acylation (Kennedy Pathway). The acyl-CoA dependent acylation is shown as the central route for TAG synthesis (Thicker line); however, Acyl CoA independent acylation has also significant role in TAG production. (**c**) Triacylglycerol structure composed of Glycerol and three fatty acids, which are attached by an ester bond (red color).

In plants, TAG is mainly produced through three sequential acylation of glycerol backbone in the glycerol pathway, which is also known as the Kennedy pathway^13^ (Fig. 1). The last step of TAG biosynthesis is the esterification of diacylglycerol (DAG), which can be proceeded through two different pathways of acyl-CoA-dependent and acyl-CoA-independent^14^. The acyl-CoA-dependent TAG biosynthesis is catalyzed by diacylglycerol: acyl-CoA acyltransferase (DGAT) enzymes using acyl-CoA as an acyl donor and DAG as an acyl acceptor to form TAG. At least three types of DGAT enzymes have been identified in plant species: DGAT1 and DGAT2, which are membrane-bound enzymes, and DGAT3 that is cytosolic (Fig. 1). The acyl-CoA-independent TAG synthesis is catalyzed by phospholipid: diacylglycerol acyltransferase (PDAT), which uses phospholipids and DAG as substrates to produce TAG^15,16^ (Fig. 1).

Although all the *DGAT* gene families and *PDAT* genes appear to be involved in TAG assembly, *DGATs* play essential roles in determining the quantity of TAG synthesis in plant seeds in comparison to *PDAT* genes^16–18^. Among the different *DGAT* gene families, *DGAT1* genes play more important roles in TAG biosynthesis and accumulation in developing seeds of soybean and Arabidopsis than other types of identified *DGAT* genes such as *DGAT2* and *DGAT3*^19–22^.

The first plant *DGAT1* gene was identified in Arabidopsis by three independent laboratories at the same time^19,20,23^. Thereafter, several homologous *DGAT1* genes have been gradually cloned and characterized in other plant species, including tobacco^24^; canola^25^; castor bean^26^; burning bush^27^; soybean^28^; peanut^29^; tung tree^30^; garden nasturtium^31^; *Echium pitardii*^32^; flax^33^; sesame^34^; and more recently in *Cuphea avigera* var. pulcherrima^35^. All these studies have demonstrated the dominate role of *DGAT1* in the determination of seed oil accumulation.

Manipulating the expression of *DGAT1* genes in Arabidopsis using Ethyl Methanesulfonate-induced mutagenesis^36^ and over-expression studies^21,23^ revealed the importance of DGAT1 as a rate-limiting enzyme in biosynthesis and regulating the quantity of seed TAG, and so seed oil accumulation. *GmDGAT1A* impacted seed TAG production in soybean seeds, while *GmDGAT2D* affected TAG biosynthesis in other tissues (mainly flowers), and response to environmental and hormonal cues^37^. In order to study the effects of *DGAT1*, *DGAT2*, and *PDAT* genes on total oil, epoxy, and hydroxy fatty acids accumulations in developing seeds, the expression levels of those genes in different plant species was evaluated^22^. The results of this study showed that *DGAT1* had the highest transcript level in comparison to the transcript levels of *DGAT2* and *PDAT* in Arabidopsis and soybean. However, in epoxy and hydroxy fatty acids accumulating plants such as castor, *S. laevis*, and *E. lagascae*, the level of *PDAT* and *DGAT2* transcripts were higher^22^. To better understand soybean *DGAT1* gene properties, full-length cDNA of *GmDGAT1A* and *GmDGAT1B* were cloned from developing soybean seeds^38^. Both transcripts were much more abundant in developing seeds than in other tissues, such as leaves, stems, roots, or flowers. While both genes showed important roles in soybean seed oil biosynthesis and accumulation, *GmDGAT1B* displayed higher expression at the later stages than *GmDGAT1A*^38^. More recently, expression analyses of soybean *GmDGAT1A*, *GmDGAT1B*, and *GmDGAT1C* found high expression levels of *GmDGAT1A* and *GmDGAT1C* in immature seeds^39^. Furthermore, constitutive expression of *GmDGAT1A* and *GmDGAT1B* in Arabidopsis led to increase in oil content at the cost of reduced protein content in the seeds. In tobacco, the silencing of endogenous *DGAT1* resulted in a reduction in quantity of seed oil while increased the accumulation of protein and sugar in transgenic lines^40^. It has been suggested that there may be an adverse relationship between the biosynthesis of TAG and the conversion of carbons into protein and sugars^40,41^.

The constitution of TAG is that of a glycerol backbone with three fatty acids that are attached by ester bonds and can be released by lipolyzing through catabolic metabolism (Fig. 1). In addition to diversity of fatty acid types bonded to the three positions in the glycerol backbone, the quantity of fatty acids determines the quality of seed oils in terms of physical, chemical, and nutritional properties. The fatty acid profiles of seed oils in soybean and other major oilseed crops are determined by five primarily fatty acids: palmitic acid (C16:0), stearic acid (C18:0), oleic acid (C18:1), linoleic acid (C18:2), and linolenic acid (C18:3)^42,43^. The manipulation of fatty acid compositions in seed oils, in order to improve the quality of oils for different purposes, has been one of the major interests and goals for oilseed crop breeders, including soybean breeders. Previous studies have shown that *DGAT1* genes may play important roles in determining the quality of acyl-CoA flux into TAG synthesis^21,36^. In the Arabidopsis AS11 mutant, in which a mutation is induced at *Tag1 or AtDGAT1* locus on chromosome II, the reduction in DGAT1 enzyme activity was associated with delayed seed development and reduced TAG formation. This reduction in DGAT1 enzyme activity was also found to be correlated with lower levels of oleic and eicosenoic acids, and higher level of linolenic in TAGs^36^. Antisense suppression of *DGAT1* in *Brassica napus* (*BnDGAT1*) resulted in increased oleic acid levels while decreased the linoleic acid content^44^. These studies have demonstrated that fatty acid profiles of seeds in oilseed crops can be affected by the expression levels or activities of *DGAT1* genes. Commercial commodity soybean cultivars have, on average, 10% palmitic acid, 4% stearic acid, 22% oleic acid, 54% linoleic acid, and 10% linolenic acid^1^. The fatty acid composition of soybean seeds oil determines its application for different food and industrial applications. For instance, elevating oleic acid content and reducing linolenic acid content in soybean oil is desirable to improve the functionality of oil and reduce the need for hydrogenation, which produces undesirable trans-fats in oils.

Although previous overexpression-based studies have shed some light on the role of the *DGAT1* gene family in soybean seed oil biosynthesis and accumulation, the current study has taken an unprecedented approach to discover the role of three endogenous *DGAT1* isoforms in soybean seed oil accumulation and composition and other seed value-added traits through simultaneous downregulation of the genes. The results of this research have demonstrated the importance of *DGAT1* genes not only on seed oil concentration, but also on other seed quality traits such as protein, sucrose, and the fatty acid profile.

## Results

### Generating transgenic lines with suppressed *DGAT1* gene expression

**Figure 2:**
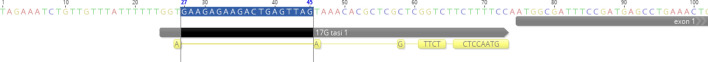
A simple scheme to show 5′UTR and start of CDS in Glyma.13G106100.1 and 17G tasi target in which match on Glyma.13G106100.

For a better understanding of the role of the *DGAT1* genes in soybean seed oil accumulation, we developed unprecedented transgenic soybean lines using trans-acting siRNA technology^45^, in which the expression of three *DGAT1* genes, (i.e., Glyma.13G106100, Glyma.09G065300, and Glyma.17G053300) which previously have been proposed in the soybean genome^46^, were simultaneously knocked down in soybean cultivar Jack. The peptide sequence of *DGAT1 (At2g19450)* gene in Arabidopsis was acquired to identify the three orthologous genes from the soybean genome using Phytozome V.12^46^. Amino acid sequences of all those three *DGAT1* isoforms, Glyma.13G106100, Glyma.09G065300, and Glyma.17G053300, showed 78%, 78%, and 68% (E-value = 0.0) identities with the *At2g19450* sequence, respectively (Table 1). To obtain the siRNA construct for suppression of endogenous *DGAT1* gene in soybean, a 422 bp fragment with homology to portions of the *DGAT1* genes was cloned behind the soybean 1514 miRNA target sequence in p1514-DGAT1-H. The silencing fragment was designed to target *DGAT1* genes Glyma.09G065300 and Glyma.17G053300 but not Glyma.13G106100. Nineteen nt was the longest stretch of homology between the silencing fragment and Glyma.13G106100 (Fig. 2).

**Table 1:** Type I Diacylglycerol O-acyltransferase (*DGAT1*) genes in Arabidopsis and soybean.

Arabidopsis gene		Soybean genes						
Name	Accession	Accession	Name	Chr	Genomic DNA size (bp)	Amino acids (aa)	Identity (%)^a^	E-value
DGAT1	At2g19450	Glyma.13G106100	GmDGAT1A	13	7840	498	78	0
		Glyma.17G053300	GmDGAT1B	17	8139	504	68	0
		Glyma.09G065300	GmDGAT1C	9	5477	394	78	0

^a^The peptide sequence resemblance of soybean candidate genes to the Arabidopsis reference genes.

**Figure 3:**
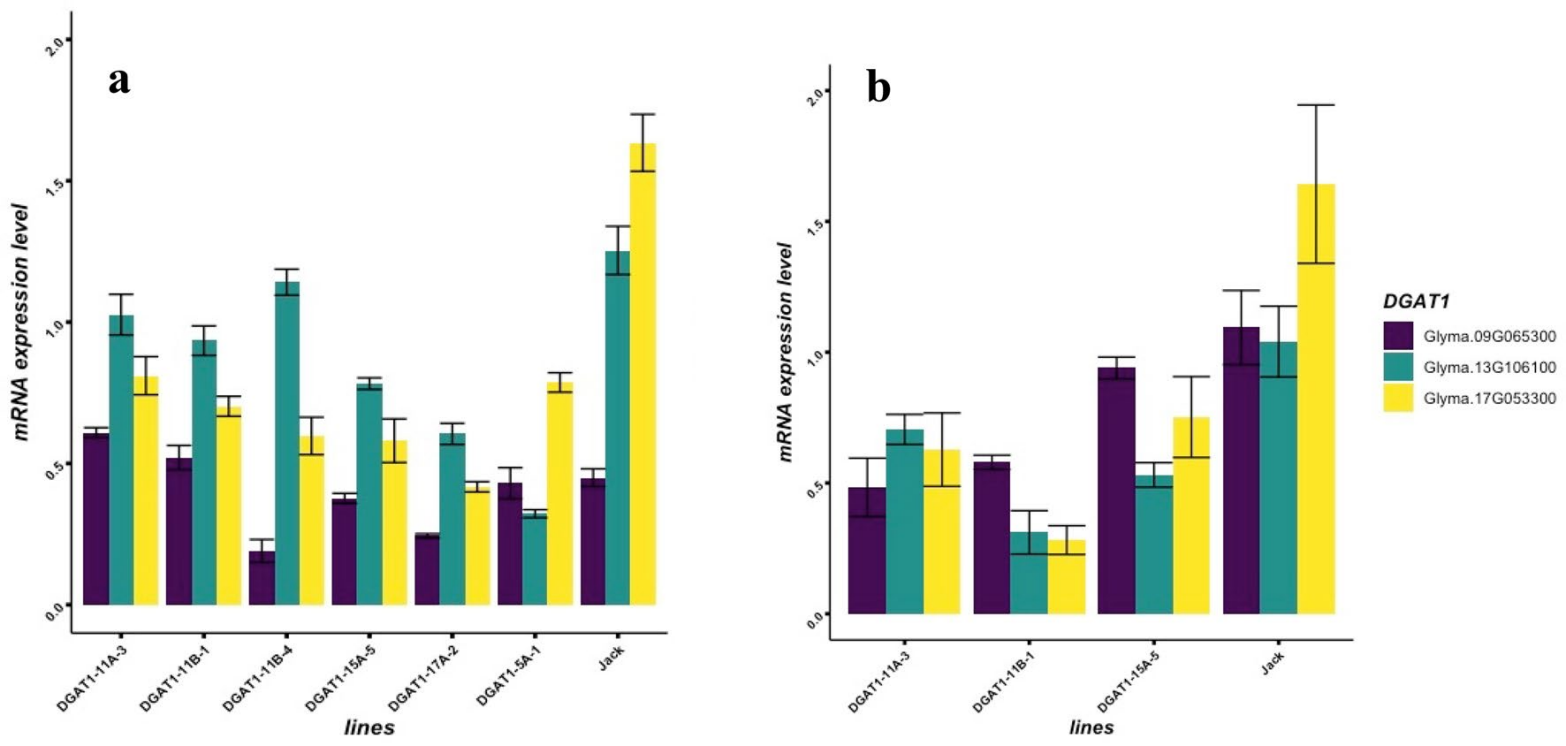
Relative expression levels of *DGAT1* genes in the wild type (cv. Jack) and transgenic soybean lines. (**a**) T_1_ DGAT1-11A-3, DGAT1-11B-1, DGAT1-11B-4, DGAT1-15A-5, DGAT1-17A-2, DGAT1-5A-1 and Jack in T_1_ generation (**b**) DGAT1-11A-3, DGAT1-11B-1, DGAT1-15A-5 and Jack T_2_ generation.

**Figure 4:**
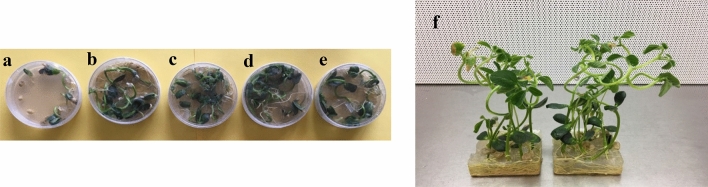
Effect of hygromycin on seed germination on transgenic and wild-type (cv. Jack) soybeans. (**a**) Wild type, in the presence of hygromycin had low germination ratios with inhibited root and shoot growths. (**b**–**e**) Transgenic soybean lines with high germination ratios and normal root and shoot growth. (**f**) Homozygote transgenic soybean after 3 weeks.

After the bombardments, transgenic lines were characterized based on hygromycin (*hph*) selection and polymerase chain reaction (PCR) identifications (Supplementary Fig. S1).To investigate the expression level of each *DGAT1* genes (Glyma.09G065300, Glyma.13G106100, and Glyma.17G053300) in the transgenic lines, seeds were collected at the R7 stage of seed development (73 days after flowering—DAF) and quantitative PCR (qPCR) analyses were conducted using gene-specific primers (Table 2). The results of qPCR showed that *DGAT1* genes expression in transgenic seeds was expressed at a very low level compared to that of the wild-type, cultivar Jack (Fig. 3). Transgenic lines with a significant reduction (P < 0.05) in the expression level of *DGAT1* genes were selected in each generation for advancing to the next generation and further evaluations (Fig. 3). Independent T_1_ transgenic lines (DGAT1-15A-5, DGAT1-17A-2, DGAT1-11A-3, DGAT1-11B-1, DGAT1-5A-1, and DGAT1-11B-4) with expression levels lower than that of the wild-type were harvested and advanced to T_2_ generation. In T_2_ generation three transgenic lines, DGAT1-11A-3, DGAT1-11B-1 and DGAT1-15A-5, that showed consistent lower expression level of *DGAT1*, than the wild-types, (Fig. 3) and were also homozygotes (Fig. 4) were selected and subjected to further analyses.

**Table 2:** Sets of qPCR (quantitative polymerase chain reaction) and PCR primers used to amplify genes specific regions and the sequences of primers and probes used for copy number assay.

Gene name	Accession	Chr. no.	Sense primer (5′–3′)	Antisense primer (5′–3′)
*DGAT1A*	Glyma.13G106100	13	AGGCAAACTTGACTGAAGGTG	TGAATTAGCCTCGCGACCTG
*DGAT1B*	Glyma.17G053300	17	GGCCTCTTCAATTCGCCTGA	TGTTTTTCGTTTTGCTGTTGGG
*DGAT1C*	Glyma.09G065300	09	CACTACCACCAGCGACTCAG	GTCGTCGTCGGTGATTTTGC
*PDATA*	Glyma.13G108100	13	TTGTTGGTTCGTGGGACTCA	TATGGCCTCCGCCACGTA
*PDATB*	Glyma.17G051300	17	TCAGCAGATGGTGGAAATGAGT	CGCACATGAAACCAGCACTT
*hph*			AGCGAGAGCCTGACCTATTG	TCGTCCATCACAGTTTGCCA

Gene name	Sense primer (5′–3′)	Antisense primer (5′–3′)	Probe	
*Hph*	CGAGAGCCTGACCTATTGCAT	CAGGCAGGTCTTGCAACGT	FAM TCCCGCCGTGCACAGGGTGT-MGBNFQ	
*Le1*	TCCCGAGTGGGTGAGGATAG	CATGCGATTCCCCAGGTATG	VIC-TGCTGCCACGGGAC-MGBNFQ

The study of exogenous gene copy number using commercially available TaqMan real-time PCR assays demonstrated nine, eight, and four copies of the exogenous gene were added into the genome of DGAT1-11A-3, DGAT1-11B-1 and DGAT1-15A-5, respectively.

### Seed composition and morphological traits analyses

**Figure 5:**
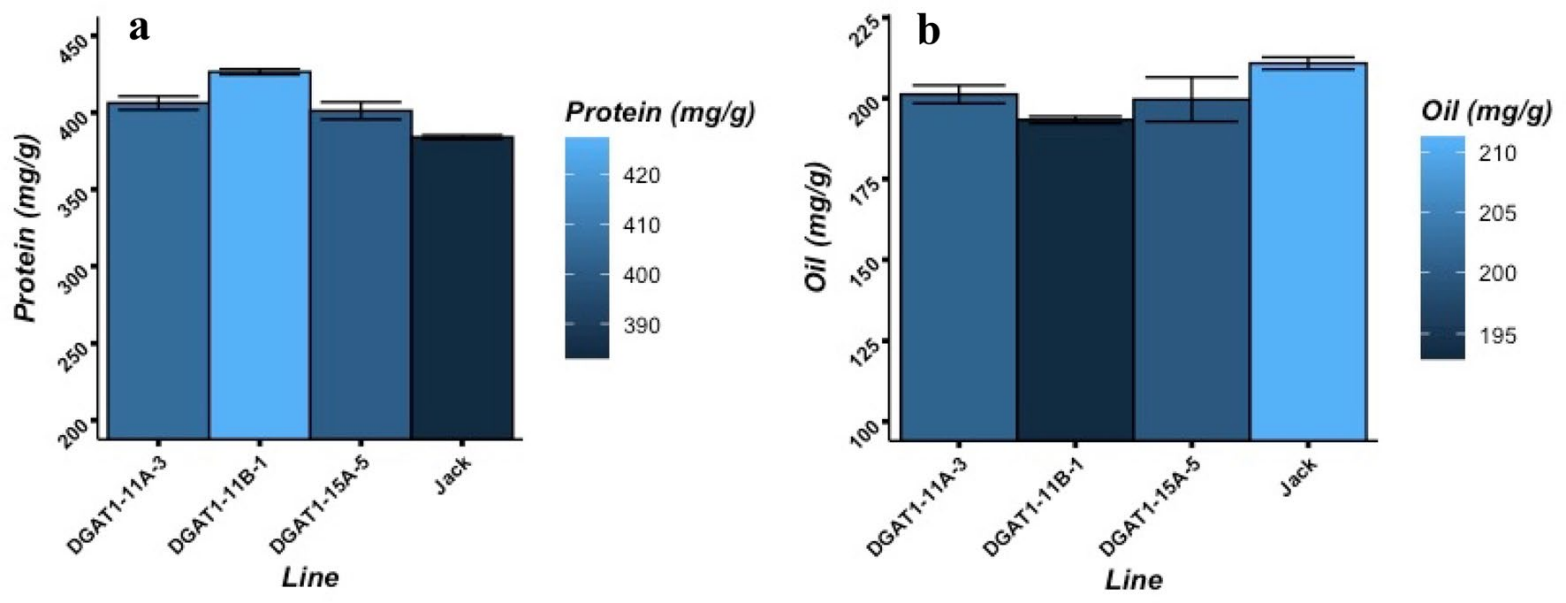
Seeds protein and oil concentrations in transgenic lines and wild type (cv. Jack). (**a**) Seed protein accumulation (mg/g). (**b**) Seed oil accumulation (mg/g). The presented values are mean ± SE measured using three independent replicates.

Seed composition traits were measured using the high‐throughput near‐infrared reflectance (NIR) method, which is now a common way of measuring seed composition traits in soybean^47,48^. The results of seed composition analyses showed that the seed oil accumulation in transgenic lines of DGAT1-11A-3, DGAT1-11B-1, and DGAT1-15A-5 were decreased by 10 mg/g (by 4.5%), 18 mg/g (8.3%), and 11 mg/g (5.3%), respectively, in comparison with the wild-type, cultivar Jack. Conversely, the seed protein concentration of these transgenic lines was increased significantly (P < 0.05) up to 22 mg/g (5.7%), 42 mg/g (11%), and 17 (4.4%), respectively, compared to cv. Jack (Fig. 5). These results were in agreement with the seed composition results attained from T_0_ and T_1_ generations that reported in Supplementary Materials, Fig. S2.

**Figure 6:**
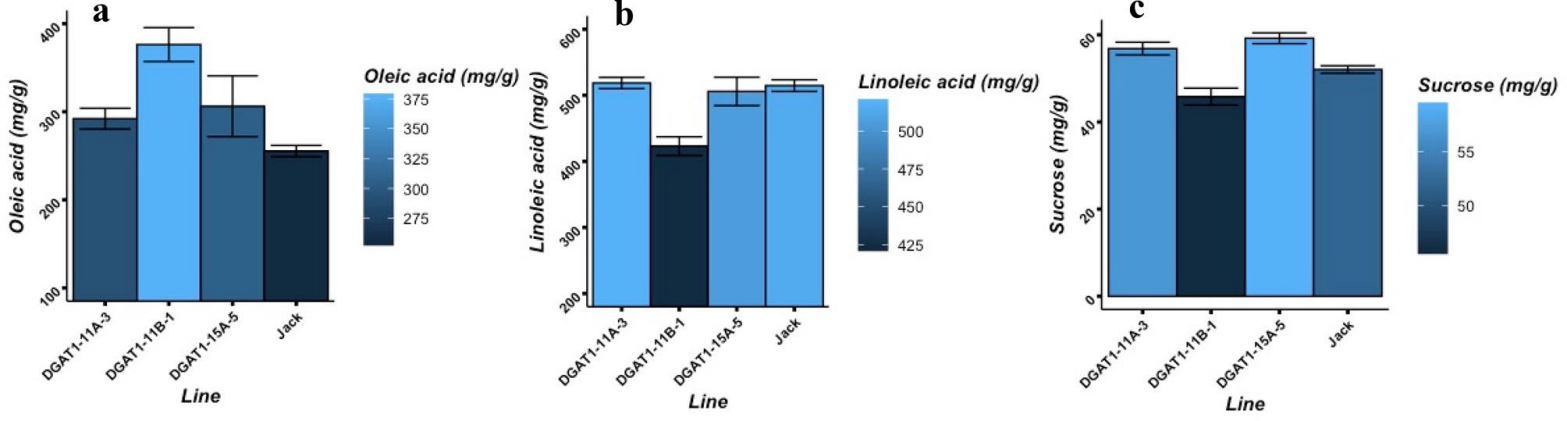
Functional impact of *DGAT1* down-regulation on fatty acids composition and sucrose concentration. (**a**) Oleic acid (C18:1) concentration. (**b**) Linoleic acid (C18:2) concentration. (**c**) Sucrose concentration. The presented values are mean ± SE measured using three independent replicates.

The fatty acid profiles and sucrose concentration in the seeds of the transgenic lines were also changed due to the suppression of *DGAT1* expressions. Profiling fatty acid compositions in the seeds showed that seed oleic acid (C18:1) concentration was increased by 37 mg/g (14.4%), 121 mg/g (47.3%), and 51 mg/g (19.9%) in transgenic lines DGAT1-11A-3, DGAT1-11B-1, and DGAT1-15A-5, respectively, compared with that of the wild-type, cultivar Jack. The seed linoleic acid (C18:2) as a major component in TAG was reduced by 91 mg/g (18%) in transgenic line DGAT1-11B-1, while no changes recorded in DGAT1-11A-3 and DGAT1-15A-5. In addition, the average seed sucrose concentration was decreased by 6 mg/g (11.9%) in transgenic line DGAT1-11B-1, while it was increased by 5 mg/g (9.2%) and 7 mg/g (13.7%) in transgenic lines DGAT1-11A-3 and DGAT1-15A-5, respectively (Fig. 6). The oleic acid increment and linoleic acid reduction in the T_2_ generation of DGAT1-11A-3, DGAT1-11B-1, and DGAT1-15A-5 transgenic lines were parallel with the results of previous generations (Supplementary Fig. S2). The values of seed oil composition in transgenic lines compare with wild-type were presented in Supplementary Table S3.

**Figure 7:**
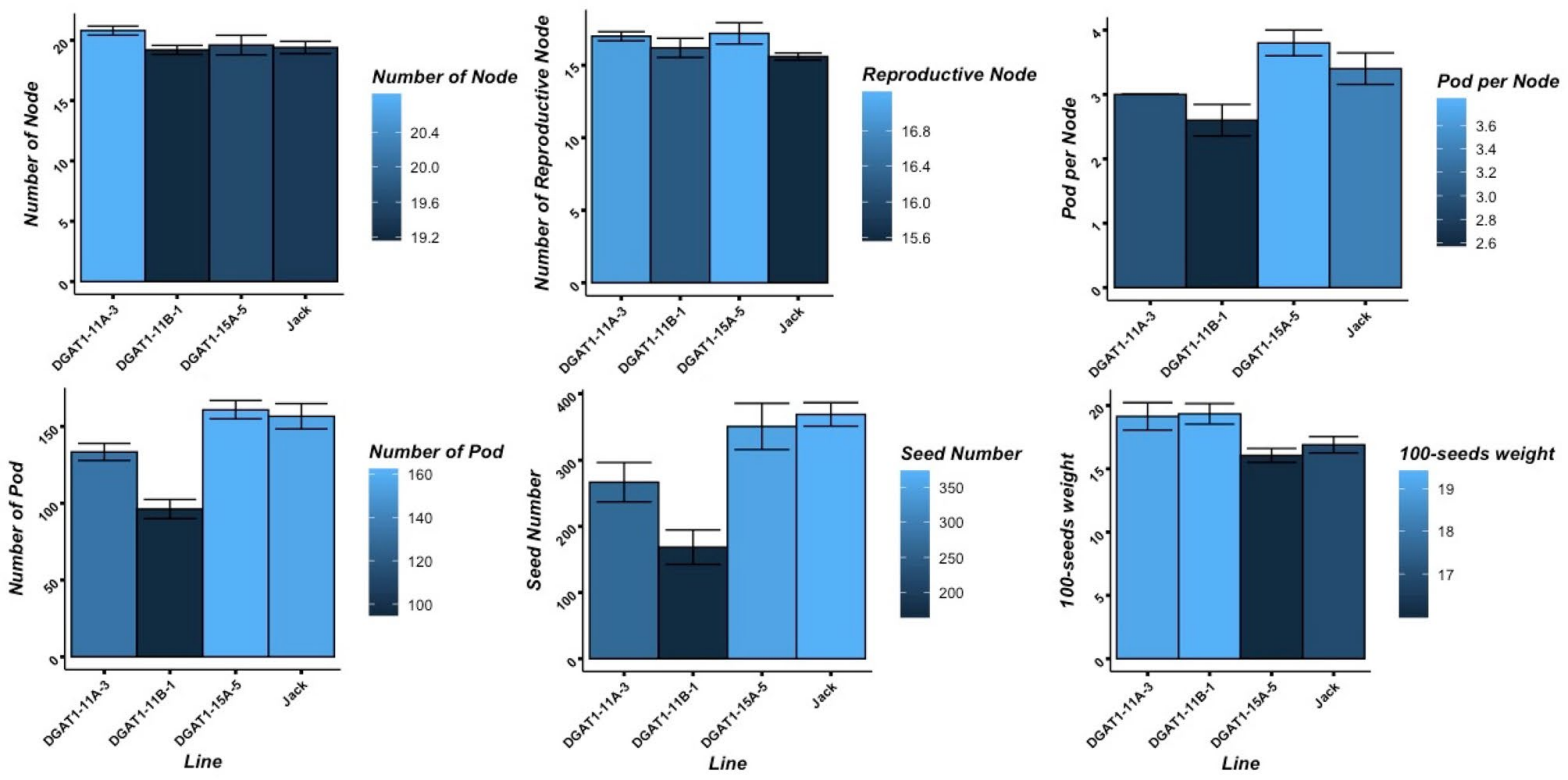
Functional influence of *DGAT1* down-regulation on morphological traits in transgenic lines and wild-type soybean, cv. Jack, at maturity stage before and after harvesting. The presented values are mean ± SE measured using three independent replicates.

The transgenic plants did not display apparent morphological changes during their vegetative growth stages in comparison to the wild-type soybean; however, some differences were observed during the late reproductive stages and pods maturity dates. Some of the morphological traits in the transgenic soybean lines seemed to be associated with differential expression of *DGAT1* genes. The number of nodes on the main stem, reproductive nodes, and pods, which typically play prominent roles in soybean seed yield production, were measured to investigate the consequence of *DGAT1* down regulation on total yield potential based on single plants. The results showed that the down regulation of *DGAT1* genes did not affect the number of total nodes and reproductive nodes on the main stem of the transgenic lines. Although it appeared that the knocking down of *DGAT1* genes had no significant effects on number of nodes, it seemed to be correlated to decreases in the number of seeds in transgenic lines DGAT1-11A-3 and DGAT1-11B-1 (Fig. 7). The reduction in number of seeds per plant in these two transgenic lines seemed to be due to the reduction in the number of pods per plants. In addition, suppression of *DGAT1* isoform seemed to be associated with a significant increase (P < 0.05) in seed weight in transgenic lines DGAT1-11A-3 and DGAT1-11B-1 (Fig. 7). None of the above morphological changes was identified in transgenic line DGAT1-15A-5 compared with that of the wild-type, cultivar Jack (Fig. 7).

**Figure 8:**
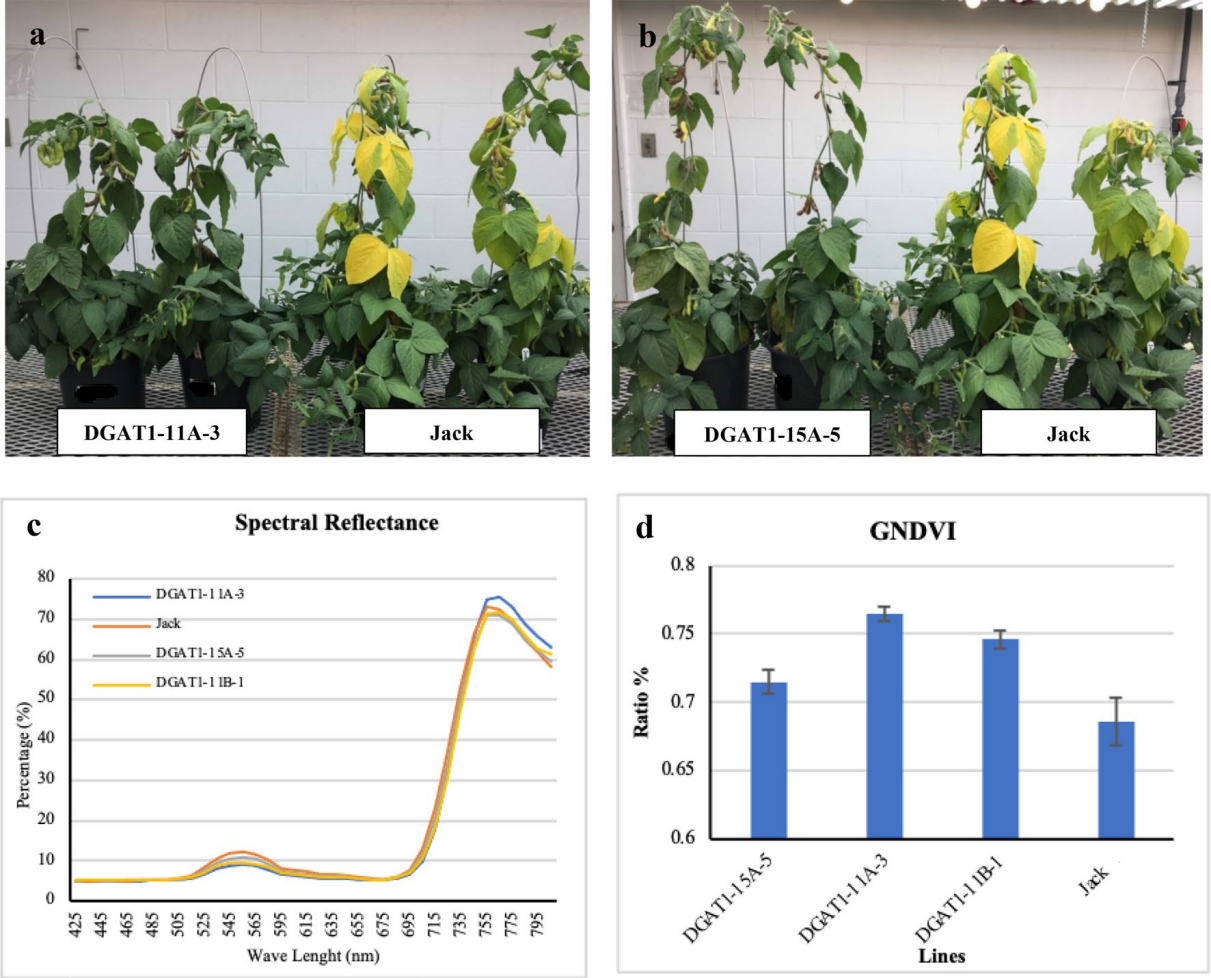
The effect of *DGAT1* down-regulation on senescence of transgenic soybean at maturity stage. (**a**) DGAT1-11A-3 (left) and wild type (right). (**b**) DGAT1-15A-5 (left) and wild type (right). (**c**) Spectral reflectance of transgenic lines and wild type. (**d**) Green Normalized Difference Vegetation Index (GNDVI). The presented values are mean ± SE measured using three independent replicates.

The difference in the progress of leaf senescence between transgenic lines and wild-type soybean at the maturity stage were noticeable. While the start of the R6 and R7 growth stages in both transgenic lines and wild-type soybeans were the same, transgenic lines showed a delay in leaf senescence. At the time that the leaves of the wild-type lines started turning yellow, the leaves of transgenic lines were completely green without any visual signs of the senescence (Fig. 8a,b). It seemed that the transgenic lines had a delay in the time of senescence onset and rate of progression. To quantify the leaf senescence variation between transgenic lines and wild-types, Green Normalized Difference Vegetation Index (GNDVI) was measured using proximal sensing. This index is sensitive to green vegetation and is calculated with the amount of near-infrared (NIR) and visible green spectral reflectance^49^. GNDVI is a modified version of Normalized Difference Vegetation Index (NDVI), which is more sensitive to the variation of chlorophyll content in the crop and a powerful index for measuring rates of photosynthesis^50^. The spectral reflectance pattern, in the range of 500 to 650 nm, in wild-type soybeans was completely different from the pattern in the transgenic lines (Fig. 8c). The senescence rate was evaluated by the value of GNDVI and significant variation (p < 0.05) was observed among transgenic and wild-type soybean lines at the first of R6 growth stage. Reduction in chlorophyll content was manifested as a decrease in GNDVI. Therefore, lower GNDVI index in wild-type as compared with other transgenic soybean lines can be a sign of enhanced senescence in the wild-type soybean plants (Fig. 8d).

**Figure 9:**
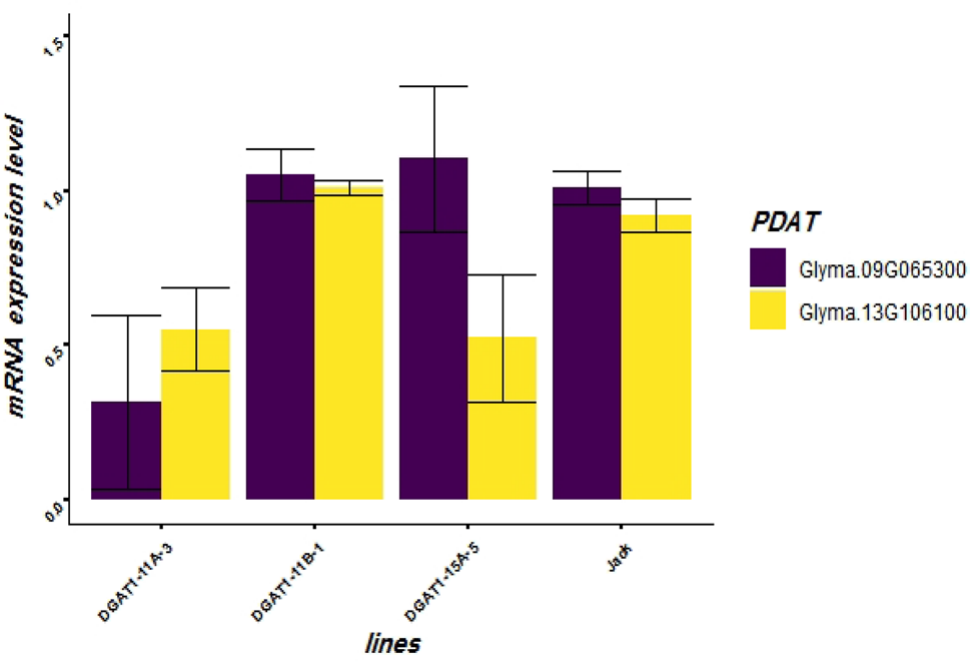
The qPCR analyses of relative expression level of two phospholipid: diacylglycerol acyltransferase (*PDAT*) isoforms in transgenic lines DGAT1-11A-3, DGAT1-11B-1, DGAT1-15A-5 and Jack at T_2_ generation.

In order to study the effects of down regulation of the three *DGAT1* genes on the activities of *PDAT* genes, the peptide sequence of *PDAT* genes (At5g13640) in Arabidopsis was acquired to identify the orthologous genes from the soybean genome using Phytozome V.12. Glyma.17G051300 and Glyma.13G108100 isoforms, both with 76.8% (E-value = 0.0) sequence identity to with At5g13640, were selected^46^. The expression levels of the two *PDAT* isoforms were quantified in transgenic lines and wild-type plant (Fig. 9). Not only *PDAT* genes were not over expressed in transgenic lines, i.e. DGAT1-11A-3, DGAT1-11B-1, and DGAT1-15A-5, to compensate for down regulation of *DGAT1* genes, but also Glyma.17G051300 had significant reduction in DGAT1-15-A-5 and DGAT1-11A-3, and Glyma.13G108100 had significant reduction in DGAT1-11A-3. No significant change in the expression level of these two *PDAT* isoforms was detected in the transgenic lines DGAT1-11B-1 (Fig. 9).

## Discussion

Due mainly to its unique composition profile, the demand for soybean seeds arises from food, feed, nutraceutical, and pharmaceutical industries. Currently, soybean seeds are an extremely valuable source of oil and protein for food, feed and different industrial purposes. As a result, developing soybean varieties with modified seed compositions that address market needs for various soybean-based products has attracted significant attention from scientists. The Kennedy pathway has been targeted as an important and promising way for manipulating the quantity and quality of TAG biosynthesis and accumulation in oilseed crops. In this pathway, diacylglycerol acyltransferase (DGAT) is the last enzyme for catalyzing diacylglycerol (DAG) to TAG and considered as an essential and rate-limiting enzyme for TAG biosynthesis. Although all the three *DGAT* gene families of *DGAT1, DGAT2,* and *DGAT3* appear to be involved in TAG assembly, *DGAT1* genes play more prominent roles in TAG biosynthesis and its accumulation in developing seeds in plants such as soybean and Arabidopsis^19–21^.

Overexpression of *DGAT1* genes significantly increased the level of oil in the seeds of diverse plant species, including soybean^9,34^, Arabidopsis^21,34^, canola and rapeseed^51,52^, garden nasturtium^31^, and maize^53,54^. While the increase in seed oil content by overexpressing *SiDGAT1* gene in soybean was intriguing^34^, the function of *GmDGAT1* in soybean was not well-defined. Many questions remain to be answered about the role of endogenous *DGAT1* genes in the accumulation and quality of oil as well as other seed composition traits in soybean seeds. Through creating soybean transgenic lines in which the three endogenous *DGAT1* isoforms are simultaneously down regulated, the current study aimed to address these questions. The results of this study demonstrate the important role of endogenous *DGAT1* genes not only in oil accumulation, but also in the quality of oil and quantity of other seed composition traits such as protein and sucrose.

**Figure 10:**
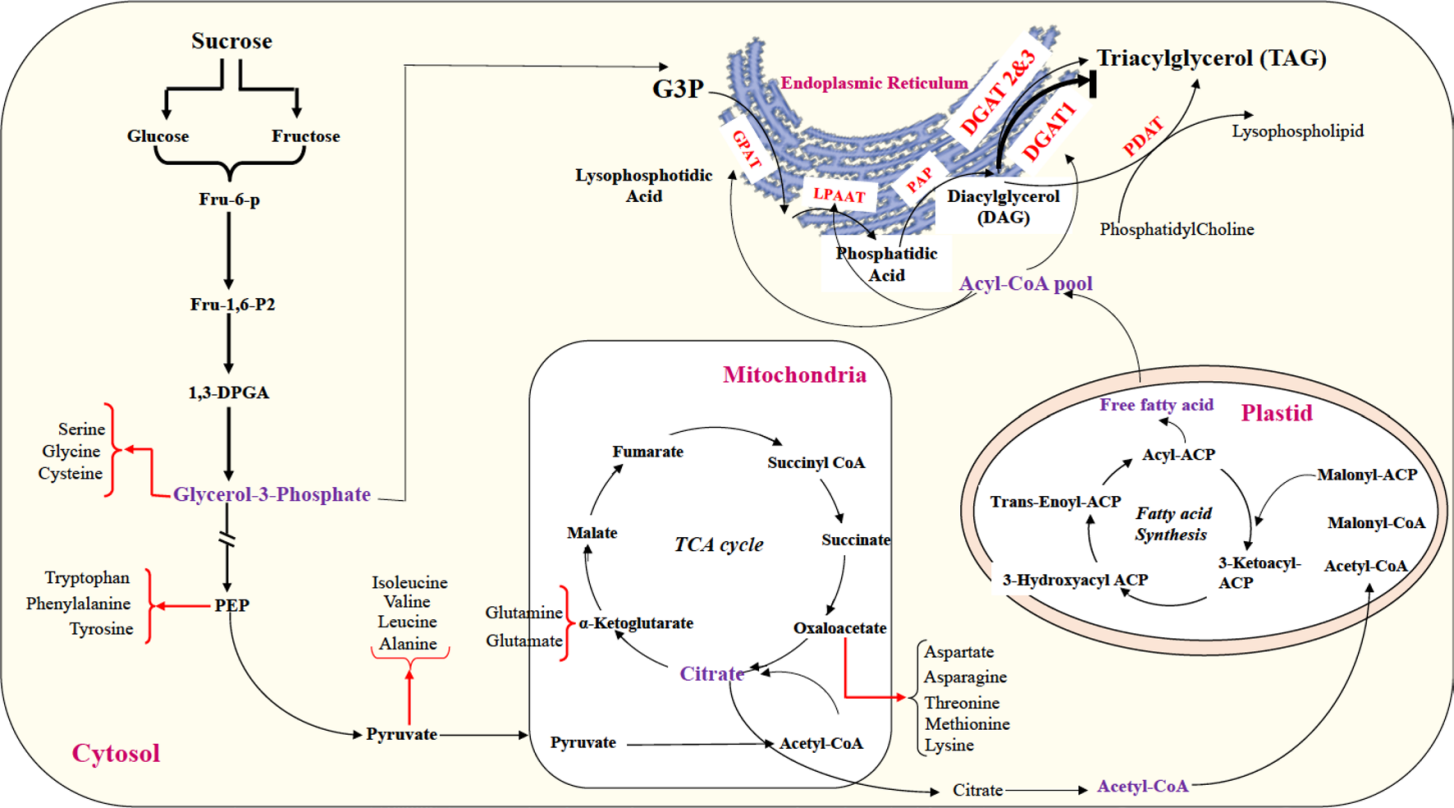
Suppression of *DGAT1* isoforms and its feedback on cytosolic glycolysis, TCA cycle, Fatty acid synthesis and Kennedy pathways products. Dash lines indicate less activity of these pathways in transgenic lines and cause the accumulation of their precursor. Red arrows indicate alternative pathways because of facing accumulation of some precursor following *DGAT1* suppression in transgenic lines. The thick black arrow indicates the higher DGAT1 enzymatic activity.

In soybean, three putative genes encoding DGAT1 enzyme have been proposed^46^, which their functions are still to be discovered and characterized. In this study, the introduction of the soybean transgenic lines with reduced expression levels of all three *DGAT1* led up to 18 mg/g (by 8.3%) decrease in seed oil with a significant increase in total protein concentration, up to 42 mg/g (11%), compared with that of the wild-type. In general, the results of this study agree with previous studies^39,40^. Previously, overexpression of three soybean *DGAT1* genes in Arabidopsis resulted in an increase in total seed oil accumulation at the expense of total protein production^39^. In another study, silencing of *DGAT1* gene in tobacco caused a significant reduction in seed oil accumulation while increased the level of seed protein contents in transgenic lines^40^. In the current study, to explain the effects of *DGAT1* genes on the accumulation of oil and protein in soybean seeds, we hypothesize that the simultaneous down regulation of the three *DGAT1* genes, which resulted in low accumulation of oil, may resulted in the accumulation of the TAG precursors, such as glycerol-3-phosphate (G3P)^55^ (Fig. 10). With less oil production in the transgenic lines, more carbon is available to flow to the protein production compared with that of the wild-type. The conversion of carbohydrate to protein as a result of a reduction in oil biosynthesis is reported in tobacco^40^.

In response to *DGAT1* genes down-regulation, the level of sucrose in the transgenic lines was also changed, but the results were not consistent among the three lines. While the level of sucrose was decreased, remarkably, in transgenic line DGAT1-11B-1, by 12%, it was increased by 9% and 14% in transgenic lines DGAT1-11A-3 and DGAT1-15A-5, respectively. Although the increased seed sucrose content was reported in the study by Zhang et al., (2005) as a result of *DGAT1* gene silencing in tobacco^40^, the negative relationship between protein and sucrose accumulation in soybean seeds is well documented in the literature^56^.

In addition to influencing seed oil, protein, and sucrose levels, our results demonstrated also that the manipulation of the expression of *DGAT1* genes may affect the fatty acid profile in soybean seeds. The downregulation of the *DGAT1* genes, in this study, resulted in a significant increase in oleic acid concentration, up to 121 mg/g (47.3%). This result is in agreement with the results of a study in Arabidopsis and soybean^37^ that showed strong negative correlations between the expression level of *GmDGAT1A* with the level of oleic acid in the seeds of Arabidopsis and soybean hairy root^37^. In another study, the overexpression of a *DGAT1* gene from sesame (*Sesamum indicum* L.) in soybean resulted in increases in palmitic and linoleic acid contents and reductions in oleic and stearic acid contents in seeds^9^. Increasing oleic acid content in soybean seed oil is considered as a desirable modification, which improves the oil’s shelf-life and its nutritional values^57^. Soybean oil typically has low oil stability and suffers from off-flavor mainly due to high levels polyunsaturated fatty acids of linoleic and linolenic^58^. By increasing the level of oleic acid in soybean seeds, the quality of the oil can be improved through increasing its oxidative stability and extending the shelf-life.

Accumulation and metabolism of TAG or oil are vital factors for activation of microspores and tapetal cells in anthers and, therefore, for male fertility^11^. TAG is an essential source of energy for pollen development and, thereby, sexual reproductions. Therefore, any changes in TAG production can affect seed production. In order to evaluate the potential effects of the *DGAT1* down regulation on seed yield productions, in this study, we compared some of yield components between the transgenic lines and the wild-type. The down regulations, while, had no significant effect on the number of flowers, nodes and reproductive nodes, it was found associated with a significant increase in 100-seed weight, and reductions in the number of pods and seeds in two transgenic lines, DGAT1-11A-3 and DGAT1-11B-1. The reduction in number of pods and seeds was positively correlated with seed yield per plant. The lower rate of the pod formation in transgenic lines, as compared to the wild-type, can be due to a down regulation of *DGAT1* genes, which is reported to be important for the grain pollen formation^11^, but levels of DGAT1 in pollen were not measured in this study. The results of this study are in agreement with previous studies that indicated seed yield of soybean to be determined by both number of flowers per plant and the proportion of the flowers that develop into mature pods^59,60^. The findings of this study are also in alignment with the results of two other studies that investigated the effects of overexpression of *DGAT1* on seeds size and yield in Arabidopsis^31,39^. In a study by Zhao et al.^39^, higher expression level of *GmDGAT1A* in Arabidopsis was correlated with larger seed size, which tended to increase seed yield. In another study, the transformation of a *DGAT1* gene from *Tropaeolum majus* into Arabidopsis showed both 1000-seed weight and total seed yield per plant to be increased in transgenic lines^31^.

**Figure 11:**
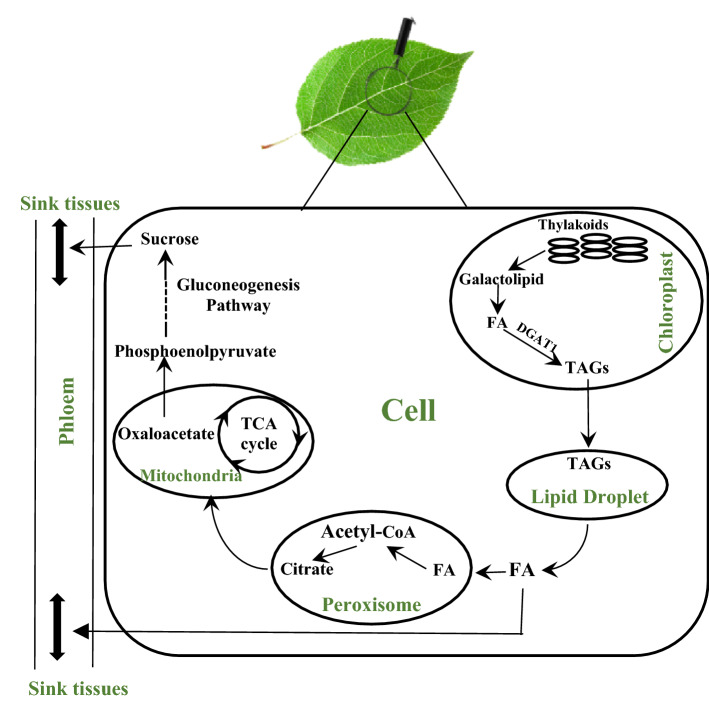
A simplified scheme of pathways involved in the turnover of membrane lipids during leaf senescence followed by translocation of nutrients to sink tissues. Galactolipases release fatty acids from Galactolipid from thylakoid membranes in the chloroplast. Peroxisome produces Acetyl-CoA and Citrate. TCA cycle in the mitochondria produces the essential precursor of Phosphoenolpyruvate through oxaloacetate. The gluconeogenesis pathway in the cytosol also converts phosphoenolpyruvate to sucrose. Phloem translocate all nutrients, including FAs and sucrose to sink tissues like seeds.

Senescence is an important time point that usually causes loss of photosynthesis activities, catabolism of macromolecules, and remobilization of nutrients to sink tissues^61^. This process is not like a programmed cell death that is induced when a given tissue is no longer needed. This phenomenon causes translocation of photosynthate from senescing leaves to other tissues such as seeds that are still in the growing or developing process^62^. Dismantling of thylakoid membranes followed by depletion of chlorophyll are two first symptoms of the manifestation of leaf senescence^63^. The membranes of thylakoids are the most important source of carbon in the form of lipid fatty acids that can be used for remobilization during leaf senescence^64^. In the senescence, galactolipid fatty acids, which are one of the most important building blocks of thylakoid membranes, are de-esterified into TAG through increasing DGAT1 activity^64,65^. Then TAG is converted into sucrose and translocated out of the senescing leaves into sink tissues like seeds (Fig. 11). In Arabidopsis, it has been also reported that *DGAT1* plays an essential role in the senescence process by sequestering fatty acids from thylakoid galactolipids into TAG^66^. It has been also observed in Arabidopsis that dominant TAG fatty acids are linolenic acid (C18:3), hexadecatrienoic acid (C16:3), and palmitic acid (C16:0) in the senescence leaves, but in young leaves, they are palmitic acid (C16:0), stearic acid (C18:0), and erucic acid (C22:1)^66,67^. This background information supports our hypothesis that indicates the down regulation of *DGAT1* has a noticeable influence on the process of senescence in transgenic lines.

In some plant species, an acyl-CoA-independent enzyme, PDAT, is involved in TAG biosynthesis or oil accumulation in seeds^16^. For example, in Arabidopsis, in a double mutant *dgat1-1* and *pdat1-1* lines, in which both *DGAT1* and *PDAT* genes were deactivated, the level of oil was reported significantly lower than the *dgat1-1* single mutant, in which only *DGAT1* gene was deactivated^10^. The findings support the importance of both *PDAT* and *DGAT1* genes for oil biosynthesis and accumulation in seeds^10^. In another study, although the *PDAT* gene was found to play an important role in seed oil accumulation in epoxy and hydroxy fatty acid accumulating plants species such as *Vernonia galamensis*, *Euphorbia lagascae* and castor, it was not reported as an influential gene for seed oil accumulation in Arabidopsis or soybean^22^. Likewise, in a study by Li et al.^22^, the *PDAT* gene did not show any effect on seed oil or fatty acid composition in an Arabidopsis mutant, in which the *PDAT* gene was knocked out^18^. The evaluation of only the two *PDAT* genes, in the current study, verified that the PDAT enzymes do not have compensating roles in oil accumulation in soybean seed. However, for a better understanding of the role of *PDAT* genes on seed oil accumulation, in the absence of *DGAT1* genes activities, comprehensive research in which all the six putative *PDAT* genes^68^ are involved is recommended.

In conclusion, this study revealed the important role of the endogenous *DGAT1* genes on the accumulation of oil, protein, and other seed compositions in soybean. While the downregulation of *DGAT1* genes resulted in decreasing oil accumulation in seeds, this reduction was not very large, which probably indicates the involvement of genes other than *DGAT1*, involved in oil biosynthesis and accumulation in seeds. In addition, significant increases of seed protein and oleic acid concentrations in the transgenic lines were among the interesting findings of this study. Based on our results, downregulating *DGAT1* genes can be exploited as a new technology for manipulating the seed composition traits in soybean seeds in favor of increasing protein and oleic acid concentrations, which are considered as important seed traits in cultivar development programs for improving the quality of oil and meal suitable for different food, feed, and industrial purposes.

## Material and methods

### DGAT1 gene-silencing construct and soybean transformation

Transgenic soybean lines have been developed using cultivar Jack (PI 540556) by trans-acting siRNA (ta-siRNA) technology^45^, in which three identified soybean *DGAT1* isoforms—Glyma.13G106100, Glyma.09G065300, and Glyma.17G053300—were knocked-down simultaneously.

For vector design, the GmUbi3 promoter:1514miRNA target:MCS:PsRbcs terminator cassette was moved from p201N 1514 via I-*Ppo*I into the same site in pSPH2^45^ to make p1514-H (Supplementary Fig. S4). The sequence (Supplementary Fig. S5) appended to the 1514miRNA target was synthesized by IDT to target Glyma.09G065300 and Glyma.17G053300 and cloned between the *Asc*I and *Avr*II sites pf p1514-H. The high similarity between Glyma.17G053300 and Glyma.13G106100 resulted in the target sequence also matching Glyma.13G106100 but only for stretches of less than 20 nt.

Transgenic soybeans were derived by Hancock et al.^69^. Briefly, translucent green immature cotyledons from zygotic embryos (≤ 5 mm) were used to induce somatic embryos on medium supplemented with 40 mg/l 2,4-D. The somatic embryos that formed were moved to medium with half the amount of 2,4-D (MSD20 medium), which was used for proliferation and maintenance. Four days before the bombardment, around 100 mg of small, compact, globular-stage repetitive embryos were arranged as a 3-cm diameter disc in the centre of a plate of MSD20 medium. Twenty minutes before shooting, the lid of the plate was opened in a laminar flow hood to allow drying of embryos. The plates were bombardment at 7584 kPa (1100 psi), 6-cm flight distance and 68.6 cm (27 in) Hg vacuum with approximately 50 ng of target DNA (p1514-DGAT1-H) attached on 667 μg of 0.6-μm diameter gold particles. Selection for transformants is done in FNL liquid medium supplemented with 20 mg L^−1^ hygromycin-B^70^. Six to eight weeks later, green clusters as transgenic candidate were selected and transferred to individual flasks.

When there was enough tissue, the presence of the transgenes was verified using a specific primer pair to *hph* (Table 2). Genomic DNA was isolated from fresh leaves of transformed and wild-type (Jack) soybean plants using NucleoSpin Plant II kit (Macherey–Nagel, Düren, Germany) as per manufacturer’s instructions. DNA quality was assessed by running samples on a 1.5% agarose gel, and quantity was evaluated using a Nanodrop spectrophotometer (ND-1000 v.3.5.2; NanoDrop Technologies, Inc., Wilmington, DE). Extracted genomics DNA was used as the template in the PCR assays. A total of 25 μL reaction mixture was prepared containing 10 ng of template DNA, 0.2 mM dNTPs, 200 nM of each primer, 3 μl 10 × Buffer and 0.2 units *Taq* polymerase (Thermoscientific DreamTaq Hot Start DNA Polymerase) using a thermal cycler (Eppendorf, Hamburg, Germany). Samples were initially denatured at 94 °C for 5 min and then subjected to 30 cycles each of 1 min at 94 °C (denaturation), 1 min at 61 °C (annealing), and 1 min at 72 °C (extension). The final extension was done at 72 °C for 10 min. The result of amplified product was visualized using 2% agarose gel.

If gene transformation was confirmed by PCR, the tissue was transferred to SHaM liquid medium for embryogenesis of cells^71^. Then the embryos were moved to MS0 medium for germination of somatic embryos. When both shoots and roots were present, they were transferred to GA-7 boxes (Magenta Corp) and eventually to soil. Transgenic plants (T_0_) were moved to the greenhouse once acclimated. The progeny of T_0_ plants are designated as T_1_, T_2_ and T_3_ and were produced in the greenhouse or growth chamber.

### Identification of homozygosity and heterozygosity in the transgenic lines

After the identification of transgenic lines, 30 seeds of each T_1_ transgenic lines were chosen to test the zygosity of plants. Seeds were surface sterilized by immersion in 5% (v/v) commercial bleaching reagent (Clorox Bleach) for 10 min after washing and cleaning seeds with tap water and one drop dish soap. Then, seeds were washed four times with sterile distilled water and were kept 5 min in the water each time. Finally, seeds were dried with sterile filter paper and sown onto autoclaved MS medium^72^ containing 1% agar and 2% sucrose and hygromycin B (GIBCO, Invitrogen corporation) at a concentration of 20 mg/L. Seeds were then kept for 4–6 days to germinate in the dark situation at 22 °C. After germination seeds were transferred to a growth chamber and incubated at 22 °C with a 16/8 h light/dark photoperiod. Green seedlings with normal root and shoot growth were considered as transgenic, while poorly germinated pale-yellow seedlings were considered as non-transgenic. The transgenic lines in which all the seeds germinated and developed normal shoot and root systems, after three weeks, were called homozygous transgenic lines.

### Copy number assay using TaqMan qPCR

All oligonucleotides for TaqMan assay were designed by Primer Express software version 3.0.1 (Applied Biosystems, Foster City, Calif.). The internal oligonucleotide probes specific for *Hph* were labeled at the 5′ end with FAM, whereas the probe specific for the endogenous gene, lectin (*Le1*)^73^ was labeled at the 5′ end with the fluorescent dye VIC. The 3′ ends of all probes were labeled with the quencher dye MGBNFQ (Table 2; Applied Biosystems, Foster City, Calif). The *Le1* gene was used as a low-copy number endogenous control for the soybean samples in the comparative Ct method.

Real-time PCR was carried out in a reaction containing 5 μl 2 × TaqMan Universal PCR Master Mix (Applied Biosystems), 500 nM of each primer, 200 nM of each probe and 1 μl of genomic DNA (15 ng) in a final volume of 10 μl. Primers specific to the *Hph,* and the *Le1* could be used together and be analyzed simultaneously within the same reaction due to having different fluorogenic TaqMan probes. Two technical replicates were performed for each sample and template-free or negative controls were set. Real-time PCR was carried out in the QuantStudio 6 flex System (Applied Biosystems, CA) utilizing the following program: 10 min at 95 °C, 40 cycles of 15 s at 95 °C, and 1 min at 60 °C. A control sample carrying single-copy of insertion was used as the calibrator sample for the copy number assay. CopyCaller Software v2.0 (Applied Biosystems) was used to analyze the copy number of the inserted gene in soybean transgenic lines according to the manufacturer’s instructions. This analysis method utilized the cycle threshold (Ct) values to extrapolate the initial concentration of target DNA in each sample.

### RNA isolation

Seeds from each soybean line were collected at the development stage of seeds or R7 stage [73 DAF (Day After Flowering), beginning of seed maturity] and were immediately submerged in liquid nitrogen and then stored at − 80 °C prior to RNA extraction. Seeds were ground in liquid nitrogen using a mortar and pestle, which were cooled down using liquid nitrogen after decontamination by ELIMINase (Decone Labs, Inc., King of Prussia, PA) and RNAse-free water. RNA extraction was performed using the Purelink RNA Mini Kit (Invitrogen, Carlsbad, CA). Approximately 100 mg of ground seed tissue was homogenized in 1.0 ml of lysis buffer containing 1% 2-mercaptoethanol using vortex to disperse the sample. Genomic DNA contamination was removed using the On-column Purelink DNase treatment (Invitrogen, Carlsbad, CA). The quality and quantity of RNA were assessed by the QIAxcel Advanced System and QIAxpert System Spectrophotometer (QIAGEN GmBH, Hilden, Germany), respectively. RNA samples were stored at − 80 °C until cDNA synthesis.

### cDNA preparation and qPCR reactions

RT-PCR was performed with the iScript Reverse Transcription Supermix (BIO-RAD) as per manufacturer’s instructions using an optimum blend of oligo(dT) and random primers to provide an unbiased representation of the 5′ and 3′ region of target genes for freedom in qPCR primer design.

The qPCR primers for *DGAT1* (Glyma.13G106100, Glyma.09G065300, Glyma.17G053300) and *PDAT* (Glyma.17G051300, Glyma.13G108100) were designed using Primer Express version 3.0.1 (Applied Biosystems, Foster City, Calif.) (Table 2) and references genes (*Cons 6* and *Cons 7*) were chosen according to Libault et al.^74^. The specificity of these primers was checked using NCBI (https://www.ncbi.nlm.nih.gov) and Phytozome (https://phytozome.jgi.doe.gov/pz/portal.html) Blast. All primers were tested for amplification efficiency by performing a five-fold serial dilution. All qPCR reactions were performed using the PowerUp SYBR Green Master Mix kit (Applied Biosystems, Carlsbad, CA). Each reaction mixture consisted of 5 μl of SYBR Green Master Mix (2X), 200 nM of each primer, 2 ng of cDNA, and nuclease-free water to bring the final reaction volume to 10 μl. The real-time reactions were performed using QuantStudio 6 flex Real-Time PCR System (Applied Biosystems, CA) with a PCR cycling protocol of 95 °C initial denaturation for 2 min; 40 cycles of 95 °C for 3 s, 60 °C for 30 s. The lack of primer-dimer or nonspecific product accumulation was checked by melt curve analysis. Analysis of gene expression results was performed using 2^−ΔΔ*CT*^ method^75^.

### Seed weight and morphological traits

Before collecting seeds of the transgenic and wild-type soybean lines at maturity, for each plant the number of nodes, number of productive nodes, number of pods, and seeds per pods were recorded for each plant. After screening and hand cleaning of seeds, seeds number and 100-seed weight were measured for each line.

Hyperspectral reflectance data at several wavelengths, ranging from 400 to 800 nm, were measured via reflectance spectroscopy using a Flame VIS–NIR Spectrometers (Ocean Optics Sensor) at the first of R6 growth stages. To reduce signal noises, three readings per plant were measured in all transgenic and wild-type soybeans. Calculation of GNDVI index was done using the following formula:

$${\text{GNDVI}} = \, \left( {{\text{NIR}} - {\text{Green}}} \right)/\left( {{\text{NIR}} + {\text{Green}}} \right)$$

### Seed composition trait analyses

On a dry weight basis, the percentages of protein, oil and sucrose concentrations of seeds and the level of fatty acids were measured using near-infrared reflectance (NIR) with a DA 7250 NIR analyzer (Perten Instruments Canada, Winnipeg, MB) with calibrations provided by Perten Instruments^76–79^. The calibration statistics for different seed composition traits, including seed protein, oil, fatty acids concentrations, were provided in Supplementary Table S6.

## Supplementary Information

Supplementary Information.
