# Supplementary material for: Effects of type I Diacylglycerol O-acyltransferase (DGAT1) genes on soybean (Glycine max L.) seed composition
Source: Sci Rep. 2021 Jan 28;11:2556. doi: 10.1038/s41598-021-82131-5 (PMC7844222; doi:10.1038/s41598-021-82131-5)
Supplement: Supplementary file 1 — Supplementary Information. [file 41598_2021_82131_MOESM1_ESM.docx]

**Effects of Type I *Diacylglycerol O-acyltransferase* (*DGAT1*) Genes on Soybean (*Glycine max* L.) Seed Composition**

Torabi, Sepideh^1^, Arjun Sukumaran ^2,3^, Sangeeta Dhaubhadel^2,3^, Sarah E. Johnson^4^, Peter LaFayette^4^, Wayne A. Parrott^4,5^, Istvan Rajcan^1^, and Milad Eskandari^1*^

*^1^Department of Plant Agriculture, University of Guelph, ON, Canada*

*^2^London Research and Development Centre, Agriculture and Agri-Food Canada, ON, Canada*

*^3^Department of Biology, University of Western Ontario, ON, Canada*

^4^ *Center for Applied Genetic Technologies, University of Georgia,* *Athens*, *GA, USA*

*^5^Department of Crop and Soil Sciences, University of Georgia, Athens, GA, USA*

Corresponding author Email: meskanda@uoguelph.ca

**Figure S1**. Screening for transgenic soybean lines using *Hph* gene as a selectable marker. An amplicon fragment of 250 bp were amplified in putative transgenic lines. **A**) PCR analyses of T_1_ generation transgenic lines: lanes 2, 3, and 5 are wild type (cv. Jack), and lanes 6 - 65 are putative transgenic lines. **B**) PCR analyses of T_2_ generation: lanes 1-18 and 24-33 are putative transgenic lines, lanes 19-23 are wild type (cv. Jack), and lane 34 is the negative control.


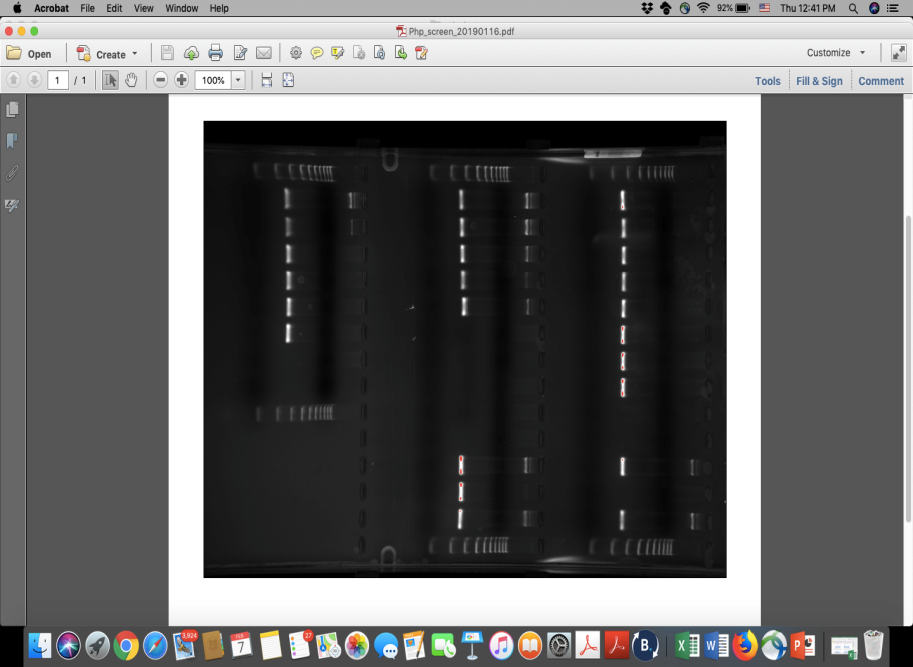


Jack

**Negative Control**

**1,000**

**100**

**200**

**300**


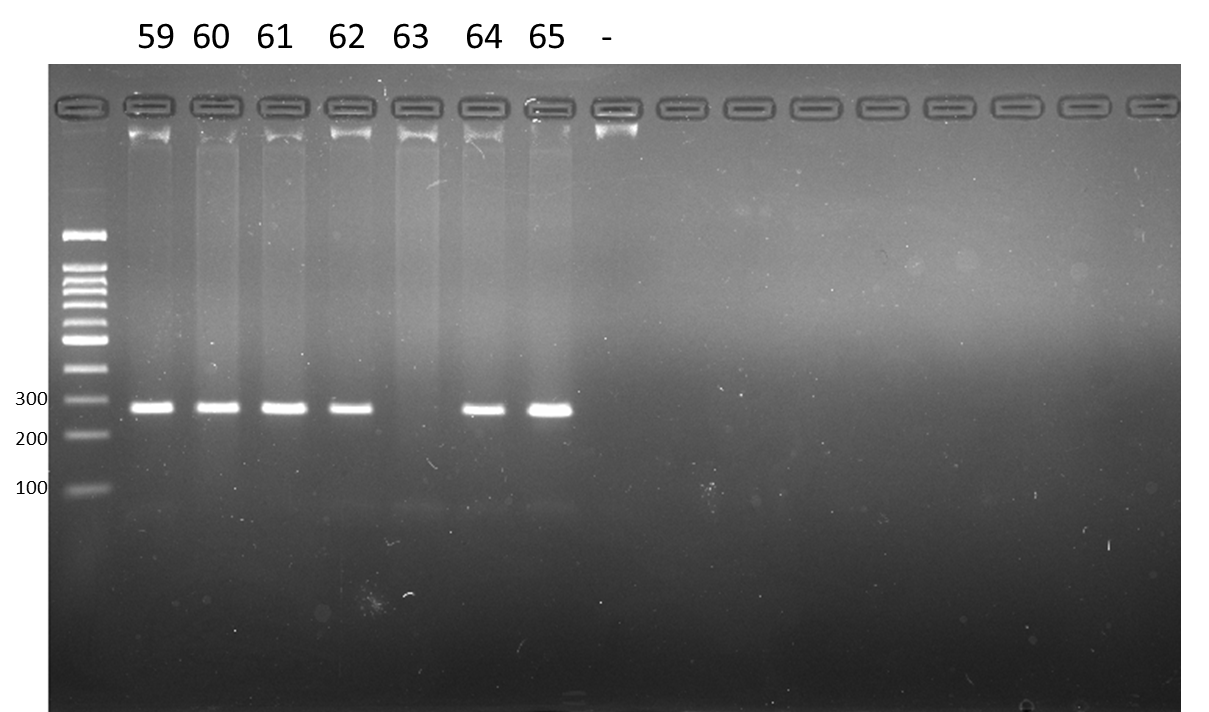

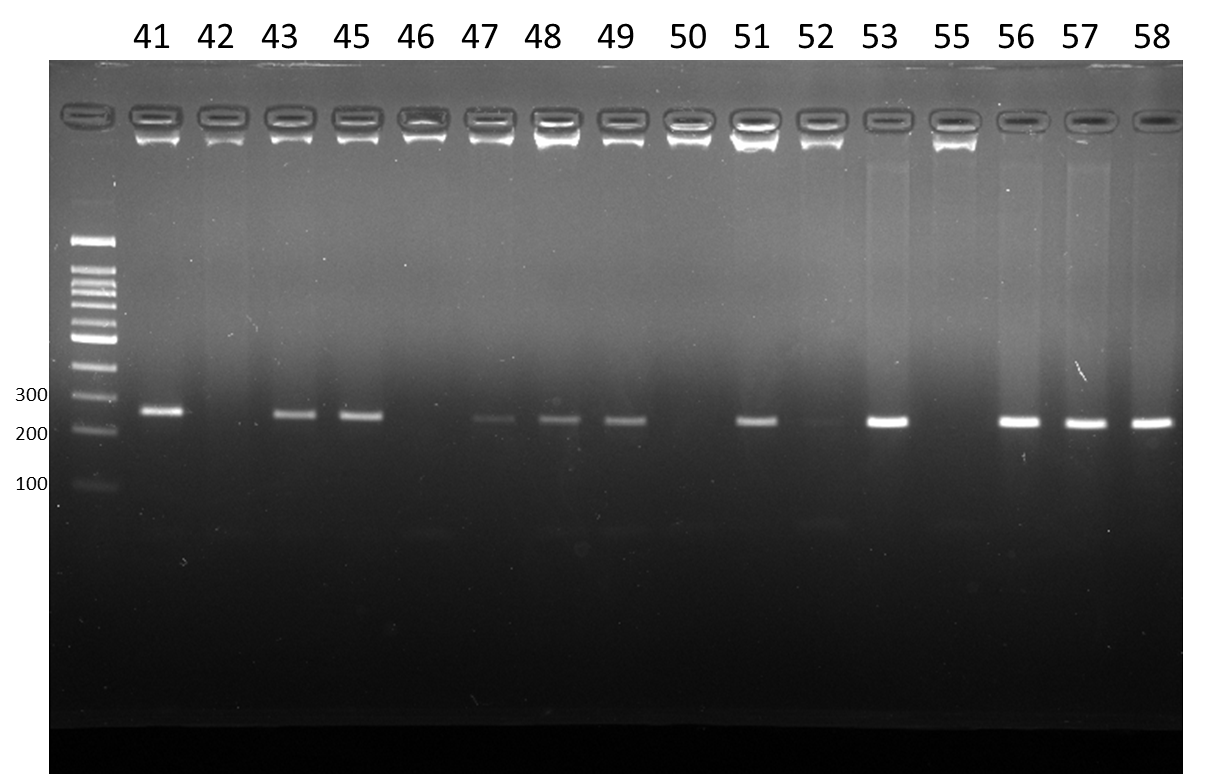

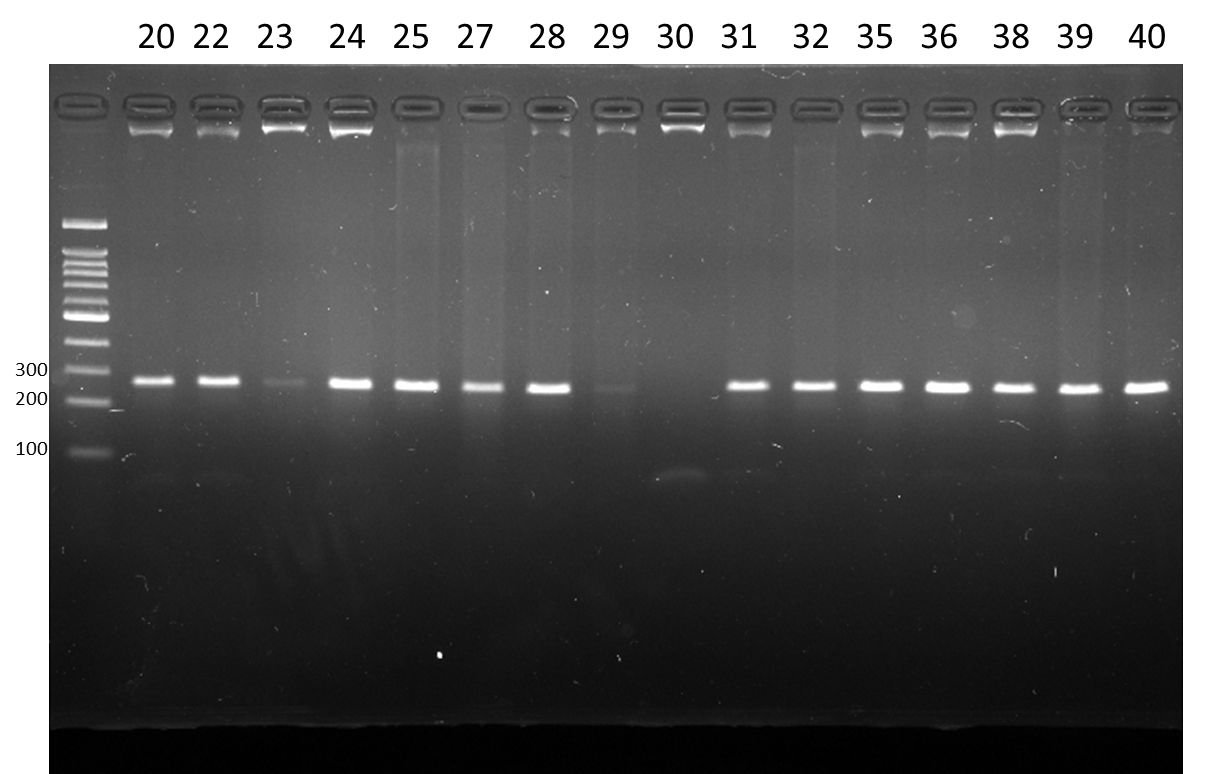

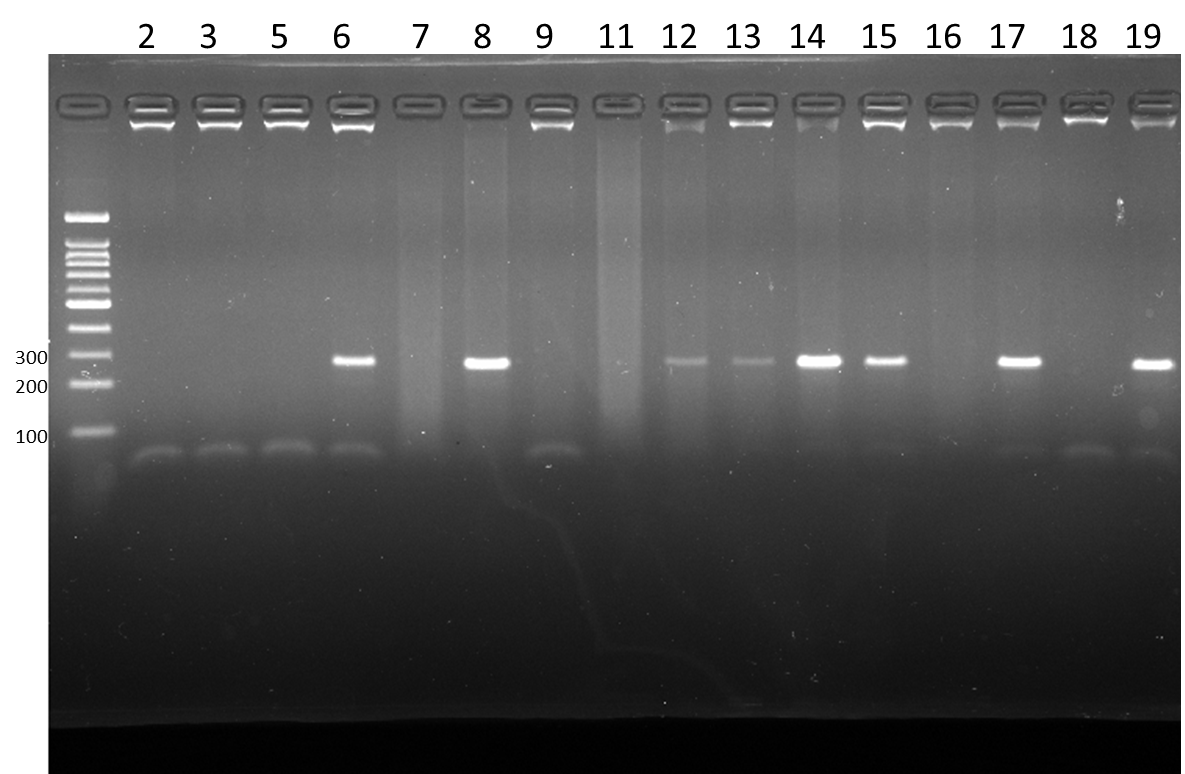


Jack

250bp

250bp

**1 2 3 4 5 6 7 8 9 10 11 12 13**

**14 15 16 17 18 19 20 21 22 23 24 25 26**

**27 28 29 30 31 32 33 34**

A

B


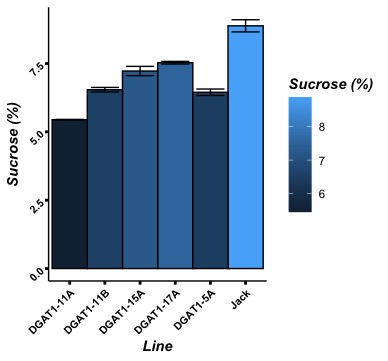

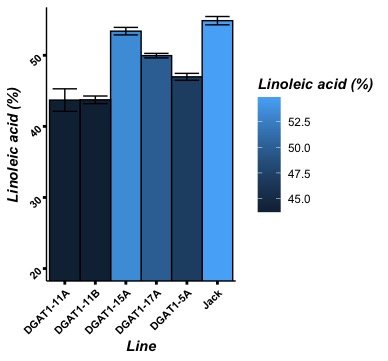

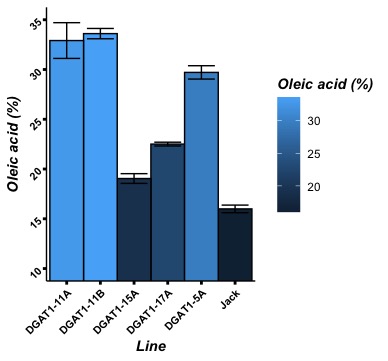

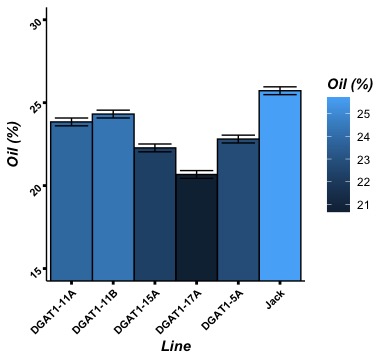

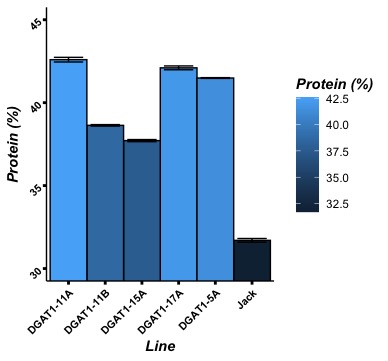


A1

A2

A3

A4

A5

**Figure S2**. Seed Oil, Protein, Oleic acid (C18:1), Linoleic acid (C18:2) and Sucrose content in T_1_ and T_2_ transgenic lines in comparison to Jack, non-transgenic lines. A1- A5) Seed Oil, Protein, Oleic acid (C18:1), Linoleic acid (C18:2) and Sucrose content in T_1_ transgenic lines B1- B5) Seed Oil, Protein, Oleic acid (C18:1), Linoleic acid (C18:2) and Sucrose content in T_2_ transgenic lines.


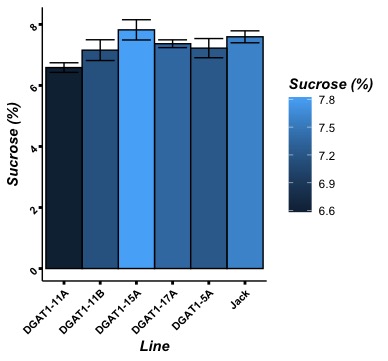

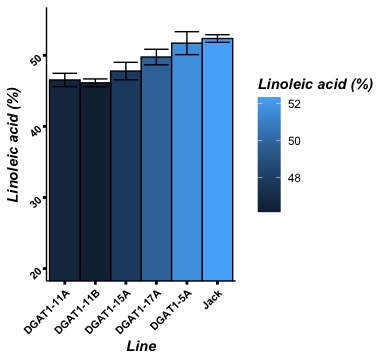

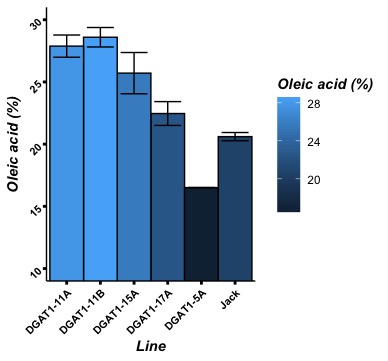

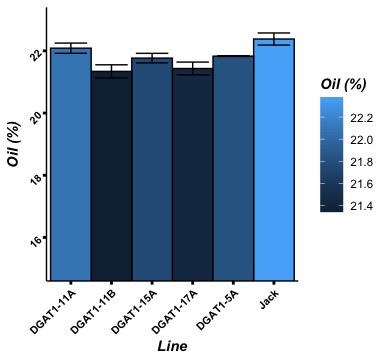

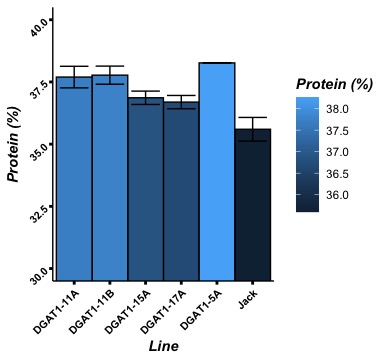


B2

B1

B3

B4

B5

**Figure S2** **(Continued**)

**Table S3**. The values of seed oil composition in transgenic lines vs wild-type

|  | **Palmitic acid %** | **Stearic acid %** | **Oleic acid %** | **Linoleic acid %** | **Linolenic acid %** |
| --- | --- | --- | --- | --- | --- |
| **Jack** | 10.78614 | 4.55528 | 25.53068 | 51.46402 | 6.7203 |
| **DGAT1-15A-5** | 9.96232 | 3.85974 | 30.6171 | 50.5877 | 5.73224 |
| **DGAT1-11B-1** | 9.56556 | 4.42812 | 37.62294 | 42.31554 | 6.5198 |
| **DGAT1-11A-3** | 10.23924 | 4.36966 | 29.2121 | 51.8604 | 5.0912 |


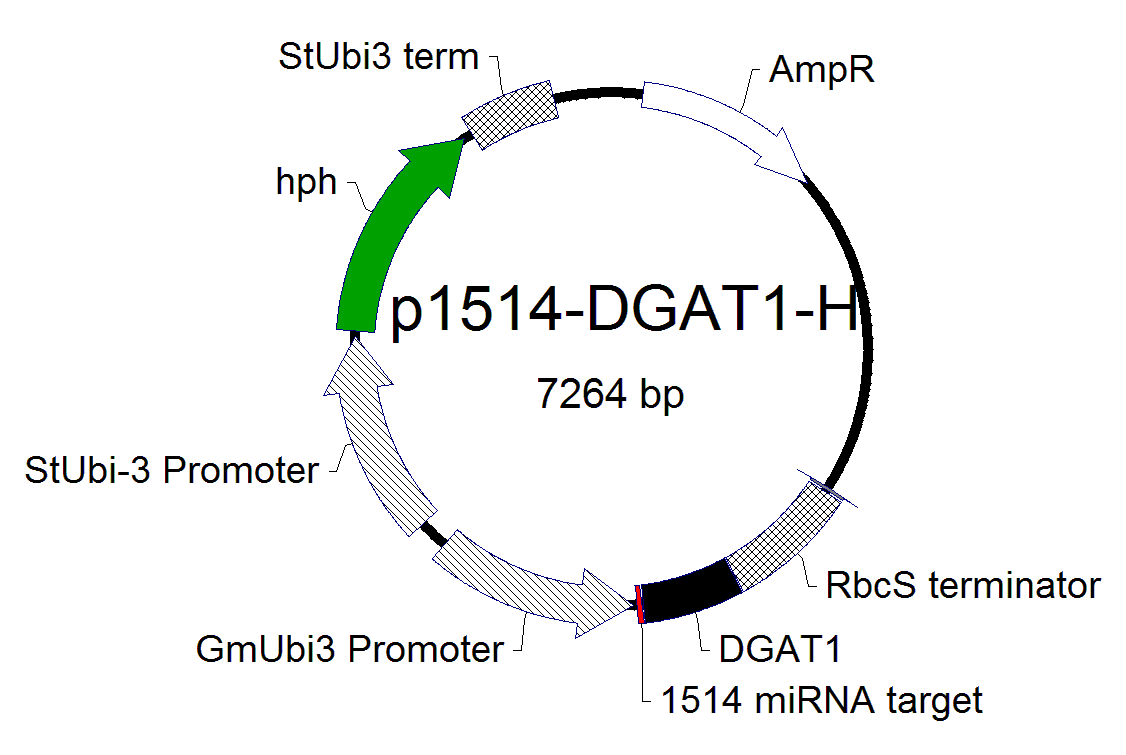


**Figure S4.** Diagram of p1514-DGAT1-H. The *hph* gene under control of StUbi-3 promotor confers hygromycin resistance and use in selection of transgenic events

ggcgcgccTGGCGATCTCCGATGTGCCTGCAGCCGCTGGCACGACCGCCACTACCACCAGCGACTCAGATCTCCGACAGCCTTCTCTGCGGCGCAGGTCCTCCGCCGGAGTCCTCTTCGACGCTGCCAGAGATTCCGGCTCCGACAATTCCCTGACCGGCAAAATCACCGACGACGACAACATCAAAGATCACAAGCCGAATAATCACGCAGCCTCCGACGACAATGTGGGCGCCGCCGCCAATGACGCTGGGCAGGAGCACCGACAACCGGggatccGGAGAAGAGAAGACTGAGTTAGAAAACACGCTCGGTCTTCTTCTCCAATGTGCGCCGCCGTCCCTCCGCCACCTCCACCGCCGGCCTCTTCAATTCGCGACTCCATCAACAGCGACGACGCCGCCGTCAATTCCCAACAGCAAAACGAAAAACAAGACcctagg

**Figure S5**. The sequences of the gBLOCK Synthetic DNA: (**Black font**) AscI, BamHI and AvrII restriction sites. (**Red font**) sequence targets of Glyma.09g07520.  (**Blue font**) sequence targets of Glyma.17g053300 with short homologies to Glyma.13G106100.

**Table S6.** Whole-seed (dry basis) calibration values for oil, protein, Moisture and fatty acid components, as provided by Perten Instruments. The coefficient of determination for cross-validation (R^2^CV) explains the proportion of variance that can be predicted between reference chemistry and predicted values. The minimum and maximum values are the limits of the prediction range. The SECV is the standard error of cross validation, where samples are removed from the validation set and predicted, and the total error for the dataset is calculated. Factors shows the number of factors included in the calibration equation for a given trait. Samples shows the total number of samples used in the calibration. Calibration file dates for each trait are listed.

| **Trait (%)** | **R^2^CV** | **Minimum** | **Maximum** | **SECV** | **Factors** | **Samples** | **Calibration File** |
| --- | --- | --- | --- | --- | --- | --- | --- |
| **Oil** | 0.83 | 13.6 | 26.5 | 0.8 | 19 | 3381 | 20170608 |
| **Protein** | 0.89 | 33.4 | 53.6 | 0.8 | 18 | 3607 | 20170608 |
| **Moisture** | 0.27 | 4.0 | 15.7 | 2.9 | 19 | 3531 | 20160609 |
| **Palmitic Acid** | 0.04 | 4.4 | 18.9 | 5.0 | 19 | 3490 | 20160609 |
| **Stearic Acid** | 0.60 | 2.4 | 6.9 | 0.5 | 19 | 3524 | 20160609 |
| **Oleic Acid** | 0.88 | 13.8 | 89.6 | 4.3 | 19 | 3423 | 20160609 |
| **Linoleic Acid** | 0.77 | 3.6 | 66.7 | 5.3 | 19 | 3434 | 20160609 |
| **Linolenic Acid** | 0.50 | 0.8 | 14.1 | 1.8 | 19 | 3514 | 20160609 |
| **Sucrose** | 0.33 | 1.2 | 10.3 | 1.2 | 20 | 1333 | 20160609 |
